# Dietary Care for ADPKD Patients: Current Status and Future Directions

**DOI:** 10.3390/nu11071576

**Published:** 2019-07-12

**Authors:** Sol Carriazo, Maria Vanessa Perez-Gomez, Adrian Cordido, Miguel Angel García-González, Ana Belen Sanz, Alberto Ortiz, Maria Dolores Sanchez-Niño

**Affiliations:** 1 Department of Nephrology and Hypertension, IIS-Fundacion Jimenez Diaz UAM, 28040 Madrid, Spain; 2 Red de Investigación Renal (REDINREN), 28029 Madrid, Spain; 3 Grupo de Genética y Biología del Desarrollo de las Enfermedades Renales, Laboratorio de Nefrología (n.°11), Instituto de Investigación Sanitaria (IDIS), Complexo Hospitalario de Santiago de Compostela (CHUS), 15706 Santiago de Compostela, Spain

**Keywords:** autosomal dominant polycystic kidney disease, diet, water, ketogenic, glycolysis, phosphate

## Abstract

Autosomal dominant polycystic kidney disease (ADPKD) is the most common genetic nephropathy, and tolvaptan is the only therapy available. However, tolvaptan slows but does not stop disease progression, is marred by polyuria, and most patients worldwide lack access. This and recent preclinical research findings on the glucose-dependency of cyst-lining cells have renewed interest in the dietary management of ADPKD. We now review the current dietary recommendations for ADPKD patients according to clinical guidelines, the evidence base for those, and the potential impact of preclinical studies addressing the impact of diet on ADPKD progression. The clinical efficacy of tolvaptan has put the focus on water intake and solute ingestion as modifiable factors that may impact tolvaptan tolerance and ADPKD progression. By contrast, dietary modifications suggested to ADPKD patients, such as avoiding caffeine, are not well supported and their impact is unknown. Recent studies have identified a chronic shift in energy production from mitochondrial oxidative phosphorylation to aerobic glycolysis (Warburg effect) as a contributor to cyst growth, rendering cyst cells exquisitely sensitive to glucose availability. Therefore, low calorie or ketogenic diets have delayed preclinical ADPKD progression. Additional preclinical data warn of potential negative impact of excess dietary phosphate or oxalate in ADPKD progression.

## 1. Background

Autosomal dominant polycystic kidney disease (ADPKD) is the most common genetic nephropathy and is a sizable contributor to end-stage kidney disease (ESRD) among those younger than 70-year-old [1,2]. Dietary intervention is a key part of the care of chronic kidney disease (CKD) patients and aims at preventing CKD progression, limiting the negative impact of CKD complications (e.g., hypertension, hyperkalemia, metabolic acidosis), or complications derived from the cause of CKD (e.g., glycemia control in diabetes) while preserving the nutritional status [3]. Some causes of CKD require specific dietary modifications, as already mentioned for diabetic kidney disease. We now review the current dietary recommendations which are common to all CKD patients and specific for ADPKD patients according to clinical guidelines, the evidence base for those recommendations, and the potential clinical impact of preclinical studies addressing the impact of diet on ADPKD progression from the point of view of the recent advances in the understanding of the pathogenesis of ADPKD.

## 2. Autosomal Dominant Polycystic Kidney Disease (ADPKD)

ADPKD is an inherited systemic disorder characterized by bilateral renal cysts that destroy normal renal tissue structure, leading to progressive loss of functioning nephrons and ESRD [4]. ADPKD is the fourth most frequent cause of ESRD, after diabetes, hypertension, and glomerular disease [5] and, thus, the most common genetic cause of ESRD [6]. The average prevalence is estimated at around 2.7 to 9.3 per 10,000 [7,8].

According to the Kidney Disease: Improving Global Outcomes (KDIGO 2012) definition of CKD, patients with ADPKD are classified as having CKD even when glomerular filtration rate (GFR) or urinary albumin creatinine ratio (UACR) are normal. Thus, CKD is defined as abnormalities of the kidney structure or function, present for >3 months, with implications for health [9]. A single criterion among the following allows the diagnosis of CKD: GFR < 60 mL/min/1.73 m^2^ or UACR ≥ 30 mg/g or abnormalities in urine sediment or tubular disorders, or pathologic abnormalities detected by histology or imaging [9,10]. Imaging allows the diagnosis of CKD caused by ADPKD even when all other parameters are normal [10]. Thus, dietary advice for CKD patients applies to all ADPKD patients, unless specified otherwise. Additionally, specific dietary interventions may provide added benefit to ADPKD patients, as discussed below.

ADPKD is characterized by enlarged kidneys in which the normal parenchyma is replaced by multiple cysts [11]. This distorts the physiological corticomedullary osmotic gradient that is required for urine concentration. Thus, urine concentration defects develop very early in the disease course, promoting polyuria, antidiuretic hormone (ADH, arginine-vasopressin, AVP) resistance and a reactive increase in AVP secretion which is best estimated by assessing copeptin, which is co-secreted with AVP but has a longer half-life [12].

ADPKD is caused by mutations in *PKD1* (which encodes Polycystin 1) and *PKD2* (which encodes Polycystin 2/TRPP2) [13,14], or more rarely, *GANAB* or *DNAJB11* [15,16]. Polycystins are crucial to maintain the phenotype of the tubular epithelium through the function of primary cilia. Defective polycystin function leads to enhanced proliferation and apoptosis, remodeling of epithelial cell membrane, and development of a secretory rather than resorptive phenotype [17]. Some of the altered intracellular signaling pathways resulting from PKD mutations were described years ago. Thus, polycystins regulate intracellular calcium homeostasis and cyclic adenosine monophosphate (cAMP) levels [11,18]. Decreased intracellular calcium and increased cAMP levels promote cell proliferation and fluid secretion, the drivers of cyst growth [18,19,20]. This information led to the development of therapeutic approaches for ADPKD. AVP increases cAMP production in tubular cystic epithelial cells through activation of vasopressin-2 receptors (V2R) [4]. Currently, the V2R blocker tolvaptan is the only approved drug for ADPKD, following the demonstration in placebo-controlled clinical trials of a protective effect against renal cyst growth, as assessed by the total kidney volume (TKV) and against the progressive loss of GFR [21]. Since AVP is secreted in response to dehydration, an increased water intake has been advocated in ADPKD patients, based on the demonstrated role of AVP in ADPKD progression and on positive experiences in experimental PKD. Additionally, somatostatin decreases cAMP production and somatostatin agonists have also been tested clinically, although with less success than tolvaptan [22,23], potentially because of the downregulation of receptors associated with cyst growth [24].

## 3. Diet in CKD

Dietary modifications play an important role in patients with non-communicable diseases [25]. The long-life expectancy in countries such as Spain, Greece, Italy, and Japan is thought to have a strong dietary influence [26,27,28]. Dietary intervention has a key role in patients with CKD and has been associated with better preservation of eGFR [25]. Therefore, different guidelines recommend considering referral to a dietitian from early stages of CKD to improve clinical outcomes [9,29,30]. In patients with advanced CKD, a diet without an optimal intake of calories, protein, sodium, and phosphate may exacerbate CKD-related clinical and metabolic abnormalities and reduce drug therapy effectiveness [31]. Thus, interventions to be considered and individualized according to patient characteristics, include reduction of protein, phosphorus, potassium, and sodium intake and limitation of the fixed acid load, with a strict follow-up to avoid malnutrition related with these restrictions. Additional consideration may relate to the cause of CKD, such as a good glycometabolic control [32].

### 3.1. Dietary Patterns

Recent data, especially from observational studies, suggest that plant-based diets, such as dietary approaches to stop hypertension (DASH), may provide benefits for patients with CKD, and delay CKD progression [33,34]. These diets are low in sodium, saturated fat, sulfur containing amino acids and phosphate and high in fiber. Thus, in opposition to meat-based diets, plant-based diets are generally alkaline-forming. Acidic food has deleterious effects on acid–base metabolism and may increase the risk of CKD progression [35,36,37,38,39]. However, larger and prospective studies are needed to confirm the advantages of plant-based diets. In any case, patients with early CKD are suggested to follow the Mediterranean diet [30]. This diet increases circulating anti-oxidant levels, which is hypothesized to be the one possible mechanism to improve survival [40,41]. The high fruit and vegetable content of the Mediterranean diet also helps reduce overall dietary phosphate intake.

### 3.2. Protein Intake

In general, patients with early CKD are recommended to avoid extremes in protein intake [42]. Low protein diets with insufficient caloric intake can lead to protein energy malnutrition. Low protein diets (≤0.6 g/kg/day) can lead to malnutrition and increased mortality [43] and high protein diets may accelerate CKD progression. Studies addressing protein intake restriction have inconsistent results, in part related to non-compliance with diet. A good example is the modification of diet in renal disease (MDRD), which was designed to provide closure to the argument over dietary protein content and CKD progression. Patients assigned to a higher protein intake did not reach the target protein intake (target protein intake 1.3 and achieved ≈1.1 g/kg/d), likely because of the spontaneous decrease in protein intake associated with low GFR. Conversely, patients assigned to the low protein intake did not reach the target (target 0.6 and achieved ≈0.7 g/Kg/d) [44]. The impact on CKD progression was marginal. Twenty-four percent of patients in the trial had ADPKD and they represented the fastest progressors. In any case, the overall evidence suggests a benefit of moderate dietary protein restriction. Current KDIGO guidelines suggest 0.8 g/kg/d of proteins in adults with GFR < 30 mL/min/1.73 m^2^ and avoiding intake >1.3 g/kg/d in adults with CKD at risk of progression [9].

By contrast, Kidney Health Australia–Caring for Australasians with Renal Impairment (KHA-CARI), 2012 Guidelines authors analyzed 5 meta-analyses, 14 randomized controlled trials, and other studies and concluded that published data have not shown consistent data that support protein restriction in early CKD. Thus, they recommended a normal protein diet of 1 g/kg/day, similar to general population [30]. Canadian Society of Nephrology 2015 recommended avoiding high protein intake (1.3 g/kg/day) in adults with CKD at risk of progression; a protein-controlled diet (0.8–1.0 g/kg/d) in adults with CKD in those with CKD G3-5, and close clinical monitoring if restricted to <0.70 g/kg/day [45]. However, the Scottish Intercollegiate Guidelines Network (SIGN) 2008 recommended protein intake <0.8 g/kg/day in early stages (G1-3), and avoiding >1.0 g/kg/day in stage G4 [46]. It was acknowledged that some studies suggest that protein restriction may be particularly effective in diseases characterized by hyper filtration, such as diabetes and certain glomerulopathies. In this regard, the recent results from the tolvaptan trials suggest that indeed hyperfiltration may be a treatable feature of ADPKD [47] although this has been disputed [48]. In any case, an adequate caloric intake must be maintained [49,50].

### 3.3. Sodium

Trials, such as the dietary approaches to stop hypertension (DASH)-sodium trial, have shown significant systolic blood pressure reduction when on low sodium diets [51,52]. This association was confirmed in systematic reviews [53]. Low sodium diets also lower proteinuria [54,55], [29,30,46]. Guidelines recommend restriction of dietary sodium intake to ≤100 mmol/day (≤2.3 g sodium, ≤6 g salt per day) in patients with CKD. As examples, KDIGO up to <90 mmol (<2 g) per day of sodium (<5 g of salt) [9] and Canadian Society of Nephrology recommends up to 65–100 mmol sodium/day. By contrast, the Institute of Medicine concluded that there is insufficient evidence to recommend a different sodium intake for CKD patients as compared with the general US population [56]. In any case, such low sodium intake is very difficult to achieve; a recent trial observed a decrease in urinary sodium excretion from around 200 to 170 mmol per day in patients randomized to a sodium intake <100 mml/day [57,58].

### 3.4. Phosphate

Hyperphosphatemia is a driver of CKD-mineral and bone disorder (CKD-MBD) and a risk factor for vascular calcification, cardiovascular mortality, left ventricular hypertrophy, and CKD progression [59,60,61,62,63]. However, phosphate restriction is not yet recommended in early stage CKD [30]. Nevertheless, serum phosphorus levels in the upper-normal range are associated with a doubling in the risk of developing incident CKD and ESRD [64].

Clinical studies addressing this issue are marred by the fact that a key source of phosphate is food additives, which is absorbed 100% in the gut, and the amount ingested from these sources is difficult to control and to quantify (reviewed in [65]). In this regard, albuminuria itself lowers the kidney production of the anti-aging factor Klotho, which promotes phosphaturia, when GFR is still normal [66]. Klotho is a co-receptor for the phosphatic hormone FGF-23, and thus, FGF-23-resistance and the consequent difficulty in excreting phosphate loading is a very early feature of CKD [67]. This would support an early intervention on dietary phosphate, but no well-design trial is available. Eventually, when compensatory mechanisms are overwhelmed, hyperphosphatemia becomes a significant problem in patients with advanced CKD [68]. KDIGO guidelines recommend to lower elevated serum phosphate levels to the normal range in patients with GFR <60 mL/min/1.73 m^2^b by using phosphate-lowering treatment and by limiting dietary phosphate intake, considering the phosphate source; the magnitude of absorption is: additives > animal protein > vegetable protein [69].

### 3.5. Potassium

Dietary potassium intake should be individualized according to serum potassium. It is not recommended to be restricted routinely, just when there is hyperkalemia [30,70]. The National Kidney Foundation Kidney Disease Outcomes Quality Initiative (NKF KDOQI) 2004 recommended a daily potassium intake of 4 g/d in CKD G1/G2, and 2 to 4 g/d for CKD G3/G4 [29]. Later, KDIGO guidelines did not recommend modification of dietary potassium intake due to insufficient evidence [9]. By contrast, current guidelines recommend potassium intake of at least 4.7 g/d in the general population, as higher potassium intake is associated with decreased cardiovascular risk [50,71].

In this regard, a recent post-hoc analysis of the modification of diet in renal disease (MDRD) study, with a sizable representation of ADPKD patients, described an association between higher urine potassium excretion and lower risk for all-cause mortality, with no association with kidney failure [72]. The potassium content of a diet is very closely linked to the amount of fruits, vegetables, and fiber discussed below.

### 3.6. Fruits and Vegetables

Patients with early CKD should consume a balanced diet rich in fruits and vegetables, as this may have a renoprotective effect and reduce blood pressure [30]. In this population, a diet with a higher proportion of plant sources (>50%) has been associated with better outcomes [50,73].

### 3.7. Dietary Fiber

The recommended daily dietary fiber intake in CKD patients is 25 to 38 g/day, the same as for the general population, as high fiber intake has been associated with lower mortality and incidence of non-transmissible diseases both in general and CKD populations, by decreasing inflammation [74,75,76]. Even a potential nephroprotective role has been suggested [77]. In this regard, fibers are emerging as nutritional components fundamental in promoting gut microbiota balance, regulating uremic toxins circulating levels and intestinal transit, and decreasing local and systemic inflammation [78].

### 3.8. Caloric Intake

Higher body mass index and central adiposity are independent risk factors for CKD progression [79,80,81]. Patients with CKD who are overweight and obese should be prescribed caloric restriction and physical activity guided by a dietitian [30]. However, the negative impact of obesity should be balanced by the increased risk of malnutrition in patients with advanced CKD. KDOQI recommends an energy intake of 35 kcal/kg/day for both non-dialyzed and hemodialysis patients younger than 60 years of age and 30 to 35 kcal/kg/d for those older than 60 years [82].

### 3.9. Fluid Intake

There is limited evidence to recommend a high volume of water intake to decrease CKD risk [83]. In a recent trial (CKD WIT), coaching to increase water intake by 1.0 to 1.5 L per day in patients with CKD did not significantly slow the decline in kidney function [84]. However, the achieved increase in daily urine volume was just 0.6 L; non-compliance was rampant despite periodic coaching [84]. CARI guidelines recommend fluid intake with moderation; in early CKD, a daily fluid intake of 2–2.5 L, including the fluid content of foods, is recommended, but emphasize that encouraging to increase fluid intake in CKD patients should not be mandatory [30]. In patients with CKD G5 in hemodialysis, higher intradialytic weight gain is associated with higher mortality risk. Thus, in this population, fluid restriction must be recommended. A key part of this recommendation is to lower salt intake, as a salty diet will promote thirst, which will make fluid restriction futile [9,42,50].

## 4. Diet in ADPKD: Current Guidelines

A key question is whether ADPKD patients should follow any specific dietary recommendations beyond the general recommendations for CKD patients of different etiologies. In general, ADPKD guidelines provide little specific advice in this regard and refer to general CKD guidelines, or advice is not based on solid data. Table A1 and Table A2 in Appendix A, summarize ADPKD guidelines and expert opinion, respectively.

### 4.1. Protein

KHA-CARI ADPKD guidelines [85] recommend a moderate protein diet (0.75–1.0 g/kg/day), as a low protein diet (<0.6 g/kg/day) has not shown to slow the rate of ADPKD progression and may increase the risk of malnutrition. The 2015 KDIGO Controversies Conference on ADPKD [86] did not recommend any specific protein intake for ADPKD patients and referred to the 2012 KDIGO guideline on CKD that recommends to lower protein intake to 0.8 g/kg/day, when eGFR is <30 mL/min/1.73 m^2^.

### 4.2. Water and Fluid Intake

Given the preclinical evidence of the therapeutic potential of AVP suppression and the clinical success of tolvaptan, an increased water intake to reduce serum osmolarity has been considered as a means to suppress AVP secretion [87,88]. However, water intake is still open to debate given the lack of clinical trials demonstrating any impact on ADPKD progression in humans.

KHA-CARI ADPKD guidelines suggest that patients with ADPKD should drink fluid to satisfy thirst, as there is no evidence that high fluid intake is beneficial for reducing cyst growth [85]. Chebib and Torres recommend moderately enhanced hydration over 24 h (including during the night if waking up) with the aim of maintaining an average urinary osmolality of <280 mOsm/kg so as to keep AVP secretion suppressed [89]. The water prescription in liters is (24-h urine solute load in mOsm/280) + insensible losses (0.5 L). Thus, optimal water intake depends critically on daily solute intake, discussed below, which is usually estimated at 10 mOsm/kg body weight but varies greatly with diet. For example, a recent general population study recorded values up to 1300 mOsm/day [90]. An individual ingesting solute to excrete 1300 mOsm/day will thus require total water intake (including in food) of (1300/280) + 0.5 = 4.6 L. Since repeat collection of such an amount of urine to assess mean 24-h urine osmolality is impractical, it is recommended to assess first morning urine osmolality, and plasma copeptin if available [89]. However, the optimal assessment will be 24-h urinary osmolality which will be in line with the recommendation by the same authors to assess 24-h sodium excretion. Di Iorio [91] indicted that the optimal fluid intake has not yet been defined.

Two ongoing or recently completed water intake trials will likely shed light on the feasibility, compliance, and impact of efforts at increasing water intake: the DRINK feasibility trial [92] and the PREVENT-ADPKD [93] trials in ADPKD [92,93].

For patients on tolvaptan, water intake is mainly driven by thirst. Thus, although preemptive water drinking is recommended, serum sodium and osmolality usually increase, indicating that patients drink according to thirst.

### 4.3. Salt

Spanish guidelines for the management of ADPKD recommend a diet with <6 g salt daily (<2.3 g/d sodium, ≤100 mmol sodium/day) to prevent and to treat hypertension similar to essential hypertensive patients and in line with general CKD guidelines [94], as do KHA-CARI ADPKD guidelines similar for early CKD [85]. The Canadian Expert consensus on Assessing Risk of Disease Progression and Pharmacological Management of ADPKD [95] recommended salt restriction similar to hypertensive subjects, according to Hypertension Canada guidelines, that is, around 5 g of salt or 87 mmol of sodium per day [96]. The guidelines specifically mention that in patients treated with tolvaptan, a sodium restricted diet of ≤2.4 g/day (≤100 mmol/day) is recommended. It is somewhat surprising that sodium restriction is again mentioned in the context of tolvaptan to emphasize similar sodium targets as in the general ADPKD population. The 2015 KDIGO Controversies Conference on ADPKD [86] also highlighted the importance of a sodium-restricted diet for blood pressure control in ADPKD. However, limitation of sodium intake may also be interesting from the osmole load point of view. Despite these recommendations and dietary instructions in the Consortium for radiologic imaging studies of polycystic kidney disease (CRISP) study, mean sodium intake was approximately 4.3 g/day (179 mmol/d) and remained constant over time [97]. Similar baseline values were observed in the HALT-PKD randomized clinical trials (RCTs) of renin-angiotensin system (RAS) blockade and blood pressure control, but in these trials sodium intake decreased to 3.5–3.8 g/d (145–160 mmol/d) during the trial and higher sodium intake was associated with faster increase in TKV or decrease in GFR in study A and B, respectively, but results were not consistent across both post-hoc analyses [98].

### 4.4. Osmole Intake

As indicated in the prior sections, osmole intake is also a determinant of the need to secrete AVP to maintain serum osmolarity. Thus, a lower osmole intake will reduce the water needed to keep AVP suppressed. A key component of the osmole load is sodium intake, which may account for 15–30% of urinary osmoles.

A low-osmolar diet in association with adjusted water intake to achieve urine osmolality of ≤280 mOsm/kg water significantly reduced AVP secretion (assessed as copeptin levels) in a small group (n = 34) of ADPKD patients followed for two weeks as compared to no intervention [99]. Patients received individually adjusted water intake to achieve urine osmolality of ≤280 mOsm/kg, and a low sodium (60 mmol, 1.5 g/day), low protein (0.8 g/kg), and low urea (i.e., avoidance of preservatives, food additives, bulking agents, and chewing gum) diet. The dietary intervention led to a reduction in water intake required to lower copeptin. However, it was likely associated with milder thirst, thus potentially keeping the effort needed to drink without thirst unchanged. In any case, patients achieved a 17% decrease in osmol excretion, urine osmolality decreased by 40% (from 426 ± 193 to 258 ± 117 mOsm/kg water) but was unchanged in the control group (from 329 ± 159 to 349 ± 139 mOsm/kg water) and copeptin levels decreased significantly by 14% vs. no change in controls. Although conceptually innovative, we believe clinical translation will be difficult given that the effort required to increase water intake to a large extent is largely replaced by an effort to change multiple dietary components, thus negatively impacting compliance. Also unanswered is whether the achieved decrease in AVP secretion is clinically relevant in humans.

Among society guidelines, osmolality recommendations were only found in the 2018 Canadian Guideline. This is the most recently updated guidelines and includes considerations about tolvaptan. The guidelines recommend that patients on tolvaptan should be referred to a dietician in order to minimize osmolal and sodium intake [95].

### 4.5. Caffeine

In cultured human PKD cells, caffeine increased cAMP accumulation [100]. However, the key question is whether the modulation of cAMP by caffeine has any clinical relevance, and what caffeine intake thresholds may be associated with clinical impact. Preclinical studies have assessed caffeine doses that are hardly clinically relevant. In murine Pkd1-deficient PKD, a high caffeine intake (150 mg/kg/d) accelerated disease progression, TKV, and decreased renal function [101], but this was not observed in rat PKD fed 400 mg/m^2^ body surface area [102]. Randomized clinical trials is needed to address the issue, but only observational studies are available that so far have not supported the clinical relevance of caffeine. In the tolvaptan era, a new question is whether caffeine intake should be a concern for patients on a drug that lowers intracellular cAMP levels.

Caffeine consumption has been evaluated in clinical studies. In a cross-sectional study, caffeine intake was not directly associated with renal volume in patients with ADPKD. The study had major shortcomings, including the cross-sectional nature, the use of ultrasound to estimate kidney volume, and low patient numbers (n = 102). In any case, caffeine intake from coffee, regular or diet soft drinks, teas, and chocolate bars was lower in ADPKD patients than in healthy controls (86 vs. 134 mg/d, approximately 1.2 and 2.0 mg/kg, respectively), likely due to prior awareness of the potential impact of caffeine restriction [103]. Thus, mean caffeine intake was just below 1 cup of coffee per day in ADPKD patients and just above that in controls. It is important to keep this perspective in mind since it implies that a high caffeine intake is uncommon in general populations. A retrospective analysis of the CRISP cohort (consortium for radiologic imaging studies of polycystic kidney disease) found no significant association between caffeine consumption from coffee, tea, and caffeinated beverages on progression of height adjusted total kidney volume (htTKV), mGFR, or time to ESRD or death in 239 patients followed for 12.5 years [104]. In the Swiss prospective ADPKD, a cohort of 151 patients was studied between 2006 and 2014. After multivariate adjustment, no statistically significantly differences in htTKV or eGFR were found between coffee drinkers and non-coffee drinkers [105].

Based on the scanty amount of good quality clinical information available, guidelines are quite conservative. KHA CARI guidelines [85] indicate that ≤200 mg/d caffeine intake (i.e., ≤2 cups of coffee or ≤4 cups of tea) could be ingested for cardiovascular health as in the general population, despite the absence of existing data associating cyst growth in ADPKD with caffeine intake. The KDIGO controversies conference for ADPKD indicated that avoiding high caffeine intake has been proposed, without defining “high caffeine” [86]. Spanish guidelines for ADPKD patients recommend avoiding drugs that stimulate cAMP accumulation, like caffeine in patients with moderate-to-severe polycystic liver disease [94]. While some guidelines do not mention other sources of caffeine beyond coffee and tea, patients should be reminded of potentially high contents in energy drinks.

### 4.6. Other Dietary Components

Guidelines do not discuss other dietary components beyond the recommendations to maintain a normal body weight as for the general population. In this regard, in the HALT PKD trial, overweight body mass and particularly obesity were strong independent predictors of TKV growth and GFR decline in early-stage ADPKD [106]. However, recent opinion pieces discuss dietary phosphate [89]. Although there are no prospective randomized studies on the effect of phosphorus in cardiovascular and renal outcomes in ADPKD, Chebib and Torres recommend a moderate daily phosphate restriction (800 mg) along with moderate protein restriction, based on observational studies in CKD discussed above [89]. While we agree with recommendations based on the excess dietary phosphate in western diets and known potential risks of excess phosphate [65], Chebib and Torres point out to an unclear rational, citing evidence that in ADPKD patients with preserved GFR hypophosphatemia is more frequent than in the general population and this was associated with high FGF23 levels and a renal phosphate leak. In this regard, FGF-23 levels are higher in patients with ADPKD than other causes of CKD at the same eGFR [107]. In this regard, in a post-hoc analysis of the HALT-PKD study, higher serum FGF23 levels did not provide independent information on the outcomes [108].

## 5. What Did We Learn from Recent Preclinical Studies?

Recent preclinical data have generated hypothesis regarding the potential impact of dietary modifications in the pathogenesis and eventual outcomes of ADPKD that should be validated in clinical studies. Areas of active research include calorie restricted and ketogenic diets, phosphate, and oxalate.

### 5.1. Calorie Restricted and Ketogenic Diets

Preclinical studies have provided interesting insights on metabolic reprogramming and glucose utilization by ADPKD cells and the impact of calorie restriction and ketogenic diets on disease progression [108].

**Figure A1:**
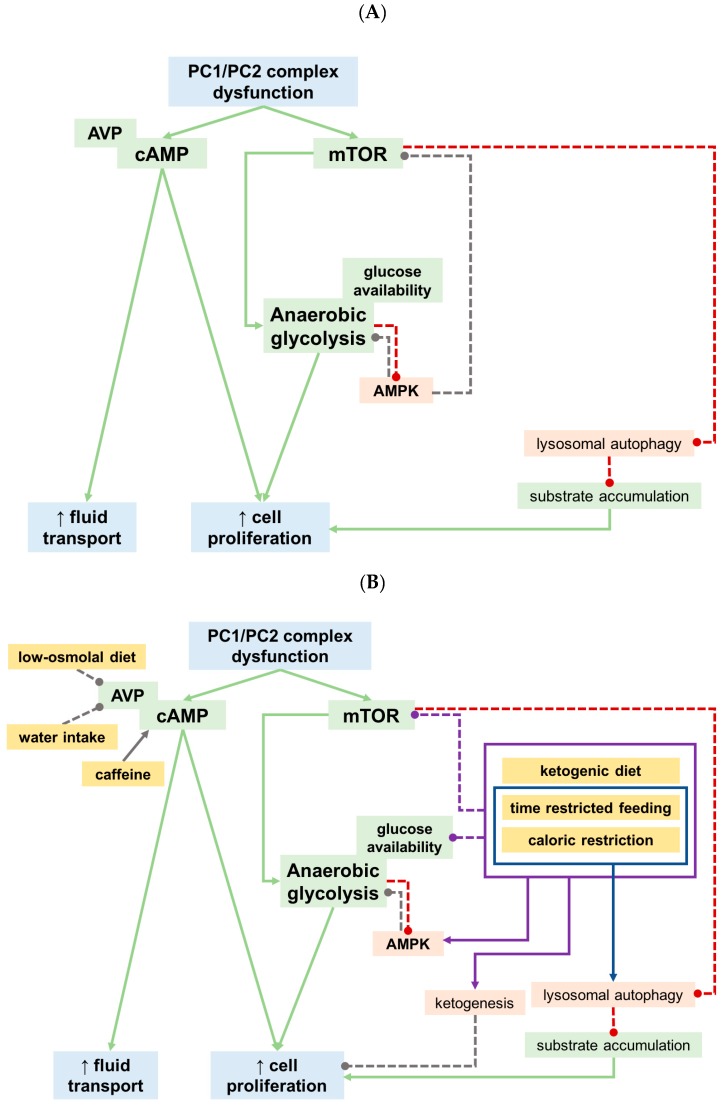

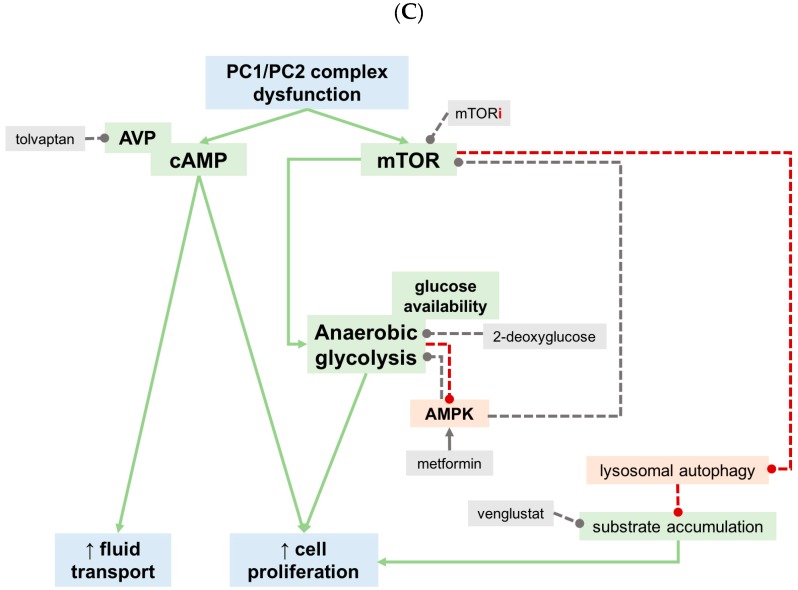
Current understanding of the molecular pathways involved in the pathogenesis of ADPKD and impact of dietary manipulation and of drugs currently in use or undergoing clinical trials for ADPKD. (**A**) Molecular pathways. Dysfunction of the polycystin-1/polycystin 2 (PC1/PC2) complex in ADPKD increases intracellular cyclic adenosine monophosphate (cAMP), which increases fluid transport into the cyst, and also increases cell proliferation which allows the cyst to increase in size. Arginine-vasopressin (AVP) further increases intracellular cAMP. PC1/PC2 complex dysfunction also stimulates the mTOR pathway that contributes to activate anaerobic glycolysis, and this favors cell proliferation and inhibits AMPK, but renders cells dependent on glucose availability. Increased mTOR activity may also disrupt the autophagy-lysosomal pathway and may increase the accumulation of certain substrates. (**B**) Impact of diverse dietary manipulation maneuvers over molecular pathways, as evidenced by preclinical cell and animal models of ADPKD. A low osmolal diet and water intake inhibit AVP, thus decreasing cAMP. A high caffeine intake may increase cAMP, but there is little evidence of clinical relevance at usual daily caffeine doses. Ketogenic diets, time-restricted feeding, and caloric restriction decrease glucose availability, inhibit the mTOR pathway, stimulates AMPK, and result in ketogenesis that decreases cell proliferation. On the other hand, time-restricted feeding and caloric restriction stimulate lysosomal degradation and autophagy. (**C**) Current therapy for ADPKD (tolvaptan) or drugs undergoing clinical trials for ADPKD. Clinical experience with venglustat, a glucosylceramide synthase inhibitor (NCT03523728) or metformin (NCT03764605, NCT02656017) has not yet been published. Blue background: structural alterations. Green background: activated molecular pathways. Red background: decreased molecular pathways. Continuous arrows: routes that activate. Red dashed arrows: pathways that inhibit. Grey discontinuous arrows: pathways that could inhibit but are not activated in ADPKD.

Glucose metabolism is altered in ADPKD renal cysts in a pattern similar to the Warburg effect found in tumors, that is, a chronic shift in energy production (ATP generation) from mitochondrial oxidative phosphorylation, to aerobic glycolysis [109,110]. The Warburg effect in epithelial cells contributed to cyst enlargement in preclinical models and targeting glycolysis might represent a novel therapeutic approach to ADPKD [109,111]. Aerobic glycolysis produces fewer ATP molecules than mitochondrial oxidative phosphorylation but is faster than oxidative phosphorylation. The faster energy production leads to a survival advantage for tumor cells, but at the same time, leads to a critical dependence on extracellular glucose levels, given the lower efficiency of anaerobic glycolysis [112,113,114]. The Warburg effect also occurs in non-tumor cells and the metabolites it generates regulate cell functions such as autophagy, apoptosis, extracellular matrix production, proliferation, and induction of some aerobic glycolysis enzymes and related metabolic markers found in non-tumor diseases, like cardiac hypertrophy, atherosclerosis, Alzheimer disease, and ADPKD [115,116]. Thus, pathways that regulate glycolysis, either inhibiting (e.g., AMP-activated protein kinase, AMPKα) or activating (e.g., mTOR) it, also modulate cyst growth. In this sense, inhibition of glycolysis by 2-deoxyglucose, activation of AMPK, or inhibition of mTOR inhibited the Warburg effect and protected from renal function loss and cyst progression in diverse preclinical ADPKD with different speeds of progression [109,111,117,118] (Appendix A Figure A1). AMPK is a metabolic sensor that negatively regulates mTORC1, and a target of metformin. Thus, the dependence of cyst cells on anaerobic glycolysis may lead to the use of novel drugs for ADPKD. A phase I RCT of 2-deoxyglucose has been designed [119] and mTOR inhibitors sirolimus and everolimus have been tested in human ADPKD RCTs [120,121,122]. However, despite success in preclinical murine *Pkd1* deficient ADPKD [123], clinical trials of sirolimus and everolimus failed to demonstrate a clinically significant benefit in human ADPKD [120,121,122]. This does not necessarily mean that dietary intervention aimed at preventing mTOR activation will fail. Thus, the pharmacological effect of sirolimus may differ from the impact of dietary modulation. Given that mTORC1 is also regulated by the nutrient supply and the energy status of the cell, this aspect can contribute to the mTORC1 activation in ADPKD [124].

In addition, the dependence of cyst cells on anaerobic glycolysis and their critical dependence on glucose availability, opens the door to attempts at dietary manipulation in disease progression. Thus, ketogenic diets, that decrease glucose availability, have been tested in preclinical ADPKD under the hypothesis that ketogenesis may be one of the factors underlying the beneficial effect of food restriction on ADPKD progression, as reduced food intake led to a partial state of ketosis, increasing levels of β-hydroxybutyrate. Indeed, in a rat model of ADPKD, a high fat, ketogenic diet led to strong ketosis and β-hydroxybutyrate elevation, and slowed disease progression, with important reduction in kidney size, proliferation, and fibrosis [125]. These results suggest that cyst cell dependence on glucose as the main energy source renders them unable to switch to the use of ketone bodies and fatty acids under conditions of ketosis and opens the door to testing ketogenic diets for ADPKD, avoiding the need for calorie restriction that presents serious compliance difficulties. Ketogenic diets induce a state that mimics carbohydrate starvation by providing high fat content (90%) and low protein and carbohydrate. There is a renewed interest in their clinical use. Guidelines recommend them for refractory childhood epilepsy [126] and they have been studied for other neurological conditions and in military training with good compliance [127,128]. Interestingly, lowering glucose availability and increasing ketogenesis is one of the hypotheses involving molecular mechanisms in the beneficial cardiorenal effects of SGLT2 inhibitors [129].

Another dietary intervention, time-restricted feeding to promote ketosis by intermittent starvation rather than caloric restriction, was also protective in a rat model of ADPKD [125]. One fourth of the world population practices a similar diet once a year during Ramadan. However, the studies on Ramadan and CKD are scarce. Only one study is available that focused on the safety of this practice and observed a decrease in proteinuria [130]. Although long ignored in ADPKD patients, albuminuria recently emerged as a risk factor for progressive loss of renal function [131]. Studies are required that explore a potential impact on long-term outcomes of ADPKD.

Prior to the exploration of ketogenic diets, calorie restriction, also a driver of ketogenesis, had shown to reduce renal cyst growth and fibrosis in preclinical ADPKD [109,110]. Mild to moderate food restriction (decrease of 30–50% of calories intake) slowed the course of the disease in murine ADPKD, without causing malnutrition. In two different *Pkd1* deficient models, reducing food intake by 40% from age 6 weeks to 7.5 months vs. ad libitum feeding reduced kidney size by 50%, decreasing the cystic index, inflammatory infiltrates, and fibrosis. Hexokinase 2 was upregulated in murine *Pkd1* deficient ADPKD and was normalized by food restriction. The magnitude was dose-dependent, and the mechanism involved was the suppression of the mTOR pathway and the activation of the liver B1/AMPK pathway. Calorie restriction had the potential to revert cystic enlargement even in older animals with progressive disease [110]. A mild reduction in food intake (23%) slowed disease progression in an orthologous mouse model of ADPKD through mTOR modulation. Reducing food intake suppressed the two main branches of mTORC1 signaling, S6 and 4EBP1 in cyst-lining cells later. Usually, the 4E-BP1 branch is poorly inhibited by sirolimus [124]. While sirolimus was previously though to completely inhibit the mTORC1 nutrient-sensitive signaling complex, mTORC1 functions that regulate cap-dependent translation and autophagy are not inhibited by sirolimus [132].

Metabolic reprogramming in cystic cells is not limited to glucose utilization. Loss of *Pkd1* in mice also changed mitochondrial metabolism, and fatty acid synthesis: *Pkd1*-mutant cells preferentially use glutamine to fuel the mitochondrial tricarboxylic acid cycle and to sustain fatty acid synthesis. Interfering with either glutamine uptake or fatty acid synthesis retarded cell growth and survival [132]. The serine-threonine kinase Lkb1 and glutaminase 1 (GLS1) are frequently dysregulated [133,134]. Both loss of Lkb1 and Pkd1 render cells dependent on glutamine for growth and inhibition of glutamine metabolism in both Lkb1/Tsc1 and Pkd1 mutant mice significantly reduces cyst progression [133]. Additional abnormalities in amino acid metabolism have been described. Histidine was preferentially used as a glucogenic amino acid favoring cyst growth [135] while branched-chain amino acids (e.g., leucine) accelerated the ADPKD progression in mice likely through mTOR activation [136]. These data provide additional avenues for dietary manipulation.

### 5.2. Phosphate

Beyond the general negative impact of phosphate excess in CKD, studies have focused specifically on the pathogenesis of ADPKD. In pcy/pcy PKD mice, a low phosphate diet resulted in slower polycystic kidney size, cystic index, and kidney fibrosis [137]. In experimental rat PKD, high phosphate diets caused calcium phosphate crystal deposition in collecting ducts, accelerating disease progression [138]. This is interesting in view of the reported higher phosphate urine clearance in early ADPKD [107]; the issue might not be as much from a systemic positive phosphate balance but from excess urinary phosphate.

Additionally, low Klotho expression was reported in kidneys from ADKD patients and in *Pkd1* knockout mice in association to Klotho promoter methylation. In these mice, treatment with recombinant mouse Klotho delayed the cyst growth [139]. While recombinant Klotho is not yet available, part of the adverse consequences of Klotho deficiency, such as accelerated aging, are linked to the inability to excrete excess dietary phosphate in urine [140]. Whether the beneficial impact of recombinant klotho in murine ADPKD is linked to protection from excess dietary phosphate remains to be studied, but this issue had obvious consequences for the dietary management of ADPKD.

### 5.3. Oxalate

ADPKD patients are at increased risk of kidney stone formation and there is recent interest of how urinary crystal formation may promote CKD progression [141,142]. In PKD rats, increased urinary calcium oxalate crystals promoted cystogenesis and PKD progression in male but not female animals [138] This finding may also have dietary implications. Thus, current dietary trends such as “juicing” which the general population may consider a healthy lifestyle to increase vegetable intake, has already been associated to kidney damage by oxalate crystals [143,144,145]. There is a distinct possibility that milder cases of juicing may promote kidney injury in ADPKD and patients should be warned of this possibility while detailed studies become available.

### 5.4. Other Dietary Approaches

By contrast, dietary enrichment by omega-3 fatty acids (flax and fish oil) or soy protein was not beneficial in preclinical ADPKD [146]. As mentioned above, PKD1 deficient mice also have defects in fatty acid oxidation, thus, changes in lipid metabolism [147]. This may be related to the fact that a C-terminal cleavage product of polycystin-1 (CTT) translocates to the mitochondria matrix. Thus, expression of CTT in *Pkd1*^−/−^ cells rescues some of the mitochondrial phenotypes [148]. Preliminary studies addressed modifying dietary fatty acids in nursing maternal mice to modulate breast milk fatty acid composition. A mild decrease in dietary soy oil (dietary fat from 6.5% to 4.0%) resulted in a smaller kidney size, although kidneys remained fully cystic [147]. The human extrapolation of these data is unclear beyond the demonstration of a potential influence of dietary fat on ADPKD progression.

## 6. Conclusions and Future Directions

There is increasing clinical and preclinical evidence that diet is a disease modifier in PKD that can be used to slow CKD progression. A key issue with dietary management of disease is compliance, especially when non-compliance does not result in obvious immediate serious adverse consequences and despite optimistic reports from a few patients followed for a few weeks [149]. As an example, although many guidelines recommend sodium restriction, the goal is highly unrealistic and usually no provision is made on how to monitor sodium intake, which would likely require 24 h urine collections. In this regard, periodic assessment of targets and providing feedback may facilitate compliance [89]. Regarding calorie restriction, the only maneuver that has increased life expectancy in all the species tested so far, the concern about compliance has led to attempts to develop drugs that mimic the effects of calorie restriction [150,151,152]. The possibility to design diverse dietary approaches that slow PKD progression, from low osmole/high water diets to food restriction to high fat ketogenic diets or time-restricted ketogenic diets may offer the variety necessary to allow switching from one regime to another and help combat monotony and enhance compliance. Additionally, active engagement of patients in guidelines development can help to create patient-centered recommendations that promote compliance [153].

So far, no dietary intervention trial specially tailored for ADPKD patients has assessed the impact on ADPKD progression. Currently, ongoing or recently completed clinical trials are only exploring an increased water intake (PREVENT-ADPKD and DRINK), both with a target urine osmolality of ≤270 mOsm/kg [92,93]. Unfortunately, based on prior trials in general CKD patients, we are not optimistic about compliance. However, there is a margin for eventual clinical success, as exemplified by trials of salt restriction which provided benefit despite rampant non-compliance with salt intake targets [57]. We should emphasize the contribution to compliance of the therapeutic relationship and on how much the clinician relies on and transmit the efficacy of the dietary manipulation. From a worldwide perspective, lack of access to drug therapy for a majority of the human population emphasizes the need to further pursue potential dietary approaches for ADPKD care. As illustrated in Figure A1A in Appendix B, these approaches may impact the very same molecular pathways that are targeted by drugs in current clinical use or undergoing clinical trials for ADPKD. Given the known physiopathology of ADPKD, potential indications for specific dietary manipulation in this context will be appropriate and should be stressed. Moreover, the fact that ADPKD diagnosis can be made even in absence of renal function decline offers the important possibility to act in a preventative way with a specific dietary manipulation, independent from the classical protein intake restriction typical of advanced CKD stages, provided that clinical trials confirm the promising results coming from preclinical studies. For this reason, the need for clinical studies in this field should be emphasized.

## Authors Contributions

All authors contributed to the literature search and critical review of the manuscript. S.C. wrote the first draft of the text and M.V.P.-G. designed the figure.

## Appendix A

**Table A1:** What do guidelines say regarding dietary management of autosomal dominant polycystic kidney disease (ADPKD)?

Intervention	Spanish 2014 [94]	Japanese 2014 [154]	KDIGO 2015 [86]	KHA-CARI 2015 [85]	Canadian 2018 [95]	The European ADPKD Forum Multidisciplinary Position Statement on ADPKD Care. [155]
Protein	NR	Limited evidence. It may be considered.	0.8 g/kg/day if eGFR <30 mL/min/1.73 m^2^ (same as non PKD-CKD), especially if total renal and liver volume is high.	(0.75–1.0 g/kg/day)	NR	Low protein diet, when appropriate (similar to KDIGO).
Water intake	High free water intake (2–3 L/d) recommended for CKD G1–3	2.5–4 L/d	Increase water intake	Drink fluid to satisfy thirst	Adjusted water intake if tolvaptan. (Goal uOSM <250 mOsm/kg.)	NR
Sodium-Salt	<6 g per day.	NR	Sodium-restricted diet.	<100 mmol/day (or 2.3 g sodium or 6 g salt per day).	According to Canadian Hypertension Guidelines: <5 g/d salt or 87 mmol/d sodium. If tolvaptan: ≤2.4 g/d (≤100 mmol/d).	Low salt diet
Osmolyte intake	NR	NR	NR	NR	Osmolyte restriction if tolvaptan to achieve a uOSM <250 mOsm/kg.	NR
Phosphate	NR	NR	NR	NR	NR	NR
Caloric Intake	Maintenance of ideal body weight.	NR	NR	Maintain healthy weight	NR	NR
Fruits/vegetables	NR	NR	NR	NR	NR	NR
Caffeine	Avoid caffeine	NR	Avoid high caffeine intake	≤200 mg/d caffeine (≤2 cups of coffee or ≤4 cups of tea per day	NR. (Coffee intake does not seem to affect adversely kidney size or function).	Control caffeine intake.
Oxalate	NR	Medical prevention not recommended because of lack of studies on efficacy.	Treat oxalate nephrolithiasis with potassium citrate.	NR	NR	NR
Ketogenic diet or fasting	NR	NR	NR	NR	NR	NR
Blood pressure target	Similar to other CKD patients.	Similar to other CKD patients.	≤140/90 mmHg. If heart disease, diabetes, proteinuria: ≤130/80 mmHg.	≤130/80 mmHg.	Target ≤110/75 mmHg if >60 mL/min/1.73 m^2^ and no significant cardiovascular comorbidity	95/60–110/75 mmHg
Acid–base	NR	NR	NR	NR	NR	NR

NR: No specific recommendations.

**Table A2:** What do opinion leaders say about dietary management of ADPKD? Recommendations about drug prescription not shown.

Intervention	Chebib et al. [89]	Di Ioro. [91]
Protein	0.8–1.0 g/kg of ideal body weight. Intervention: Dietitian counseling, monitoring protein intake: 6.253 (urine urea nitrogen in g/d1(0.033 weight in kg)).	No clear evidence on low-protein diet delaying ADPKD progression. Any recommendations based on non–ADPKD CKD patients. If prescribed, also should be accompanied with adequate energy intake.
Water intake	Enhanced hydration over 24 h. Water prescription according to urine osmolality. (24 h urine solute load (mOsm)/280) + 1 insensible loss (~0:5 L). Measure 24 h-urine sodium and first morning urine osmolality, plasma copeptin if available.	No evidence about efficacy No studies on deterioration of residual renal function. Useful in preventing renal calculus. Could be recommended in early stages of the disease, but not if moderate to severe reduction of renal function.
Sodium-salt	Guided by counseling and/or dietitian follow-up. Moderate restriction (2.3–3 g/d), adjusted for renal losses if appropriate (hot climate, runners, sauna, bowel disease)	Dietary sodium restriction.
Osmolyte intake	Patients adherent to moderate sodium and protein restrictions will have lower osmolar loads.	NR
Phosphate	Goal: Moderate intake. 1. Phosphate restriction (800 mg/d), with dietitian counseling 2. Read food labels and watch for foods additives with phosphates.	Follow dietary recommendations that patients with non-ADPKD CKD.
Caloric Intake	Goal: Normal BMI (19–24.9 kg/m^2^). Moderate caloric intake. Intervention: Dietitian follow-up and regular exercise.	Adequate caloric intake if low protein diet.
Fruits and vegetables	Increase fruits/vegetables (2–4 cups/day)	If protein restriction, increase fruit and vegetables intake, and limit animal protein.
Caffeine	NR	NR
Oxalate	NR	NR
Ketogenic diet or fasting	NR	NR
Hypertension	Blood pressure control: DASH-like diets in early stages	NR
Acid-base	Goal: Plasma HCO3 in normal range. >22 mEq/L. Increase fruits/vegetables (2–4 cups/d)	Correction of metabolic acidosis. A vegetarian low-protein diet makes possible a 50% decrease of administered sodium bicarbonate.
Lipid control	Goal: LDL ≤ 100 mg/dl. Intervention: 1. Dietician. 2. Regular exercise.	NR
